# Pennslyvania—Proposed Act to Establish “the State Live Stock Sanitary Commission of Pennslyvania”

**Published:** 1894-12

**Authors:** 


### PENNSYLVANIA.

### Proposed Act to Establish "The State Live Stock Sanitary Commission of Pennsylvania" and to Provide for the Control and Suppression of Tuberculosis and other Dangerous Diseases of Domestic Animals.

Section 1.—Be it enacted, etc.

That a Commission is hereby established to be known as "The State Live Stock Sanitary Commission." This Commission shall consist of five Commissioners, to be appointed by the Governor, as follows: the Secretary of the State Board of Agriculture, a practical breeder of live stock, the Secretary of the State Board of Health and two competent and qualified veterinarians who are members in good standing in any legally chartered veterinary medical association of this State, or who are graduates in good standing of some recognized and reputable veterinary college. Each Commissioner shall be appointed for a period of four (4) years, and shall be subject to removal by the Governor for incompetency or neglect of duty.

Sec. 2.—It shall be the duty of the Commission to protect the health of the domestic animals of the State; to determine and employ the most efficient and practical means for the suppression, control or eradication of dangerous, contagious or infectious diseases among the domestic animals, and especially of tuberculosis: and for these purposes it is hereby authorized and empowered to establish, maintain, enforce and regulate such quarantine and other measures relating to the movements and care of animals and their products, the disinfection of suspected localities and the destruction of animals as it may deem necessary.

Sec. 3.—Whenever it shall be adjudged necessary to the interests of the live stock industry of this State or to the welfare of the public to kill an animal or animals suffering from some contagious or infectious disease and an agreement cannot be made with the owner or owners thereof as to their value, three appraisers shall be appointed, one by the owner or owners of the cattle, one by the Commission or a Commissioner, and a third by these two, who shall under oath or affirmation appraise the animal or animals at their actual value at the time of the appraisement, and the owner shall be paid one-half of the said appraisement; provided that no such appraisement shall exceed fifty dollars for single unregistered cattle, or seventy dollars for single registered cattle, or forty dollars for a glandered horse or mule ; and further, provided, that no animal or animals found to be infected with contagious or infectious disease within ninety days after having been brought from another State into Pennsylvania shall be paid for, but, notwithstanding, the Commission shall be authorized to slaughter such animal or animals if, in their judgment, such a course is necessary to the interests of the live stock industry or to the welfare of the public ; and provided, that in case it shall be found, upon making the autopsy of an animal appraised and killed by order of the Commission or of a Commissioner, that such animal was not at the time of slaughter suffering from the contagious or infectious disease supposed to have been present, the entire appraised value shall be paid to the owner or owners.

Sec. 4.—The Commission, or any Commissioner, or any of their duly authorized agents shall at all times have the right to enter any premises, farms, fields, pens, buildings, cars or vessels where any domestic animal is at the time quartered, or wherever the carcass of one may be, for the purpose of examining it in any way that may be deemed necessary to determine whether they are or were the subjects of any contagious or infectious disease.

Sec. 5.—Any person or persons wilfully violating any of the provisions of this Act, or any of the regulations of the "Live Stock Sanitary Commission," or wilfully interfering with officers appointed under this Act, shall be deemed guilty of misdemeanor and shall, upon conviction, be punished by a fine not exceeding one hundred dollars, or by imprisonment not exceeding one month, or both, at the discretion of the Court.

Sec. 6.—The Commission is hereby empowered to employ such assistants and agents, and to purchase such supplies and materials as may be necessary in carrying out the provisions of this Act, and to administer oaths or affirmation to the appraisers appointed under this Act.

Sec. 7.—The salaries of the Commissioners shall be fixed each year by the Governor.

Sec. 8.—For the purpose of this Act the sum of $50,000 annually is hereby appropriated.

Sec. 9.—This Act shall go into effect July 1st, 1895, and all Act or parts of Acts inconsistent herewith are hereby repealed.

The subject of herd inspection and the extermination of contagious diseases among our domestic animals has excited a great deal of attention of late and has been the subject of much discussion Farmers realize that there is a prejudice against milk from uninspected cattle that is extensive enough to greatly restrict their market. It has been shown by Mr. George Abbott that the amount of milk consumed in Philadelphia has remained stationary during the last three years, whereas before that time there was an annual increase of several million quarts in the amount consumed. This is due, no doubt, to the agitation that has been carried on by the newspapers in regard to impure milk and diseased cattle, and to the constantly growing sentiment among consumers in favor of milk from cows that are known to be free from tuberculosis and other diseases. Moreover, tuberculosis has been becoming more prevalent among cattle during the past few years; live stock breeders are more fully appreciating the dangers of the disease and there is a general movement among them to demand an inspection of their herds by and at the expense of the State, and payment for the tuberculous animals that are found. The physicians of our cities are also in favor of some legislation that will tend to increase their confidence in the milk supply.

This demand for dairy inspection comes, therefore, both from the country and the city, so that it is practically unanimous, and there can be but little doubt that the coming legislation will provide methods and funds for this much desired measure. The many farmers who have suffered extensive losses from the ravages of tuberculosis are especially desirous of legislation that will exterminate this disease and, in part, reimburse them for the losses they are sustaining. A law which is acceptable to all of the interests concerned has not, until now, been presented to the public.

Quite recently a number of men representing the farmers and the veterinary and medical professions have been brought together in conference from all parts of the State to draw up an act that will fully cover the ground and will be just and equitable to all concerned. The result of their labors is attached hereto.

After much study and consultation it was discovered that it would be necessary to place the control of this matter in the hands of a commission in which the milk and meat consuming public, as well as the farmers, would have absolute confidence. This has been reached in the proposed "State Live Stock Sanitary Commission." It will be noticed that the composition of the commission is broad, but not broader than its work or the influence that it will be necessary for it to exert to yield the best results to the live stock owners. There are no oppressive features in the act, and under the direction of a discreet, conservative, scientific and practical commission it will surely yield the desired results.

The appropriation asked for is $50,000, but this is very modest when it is remembered that the interests to be guarded are among the most important in the State ; that they have an important relation to public health and that the market for milk and other dairy products will, no doubt, be increased far more than this amount during the first year of the operation of the law.

